# Enhanced gene expression from retroviral vectors

**DOI:** 10.1186/1472-6750-8-19

**Published:** 2008-02-25

**Authors:** Magnus Blø, David R Micklem, James B Lorens

**Affiliations:** 1 Department of Biomedicine, University of Bergen, Bergen, Norway

## Abstract

**Background:**

Retroviruses are widely used to transfer genes to mammalian cells efficiently and stably. However, genetic elements required for high-level gene expression are incompatible with standard systems. The retroviral RNA genome is produced by cellular transcription and post-transcriptional processing within packaging cells: Introns present in the retroviral genomic transcript are removed by splicing, while polyadenylation signals lead to the production of ineffective truncated genomes. Furthermore strong enhancer/promoters within the retroviral payload lead to detrimental competition with the retroviral enhancer/promoter.

**Results:**

By exploiting a new method of producing the retroviral genome *in vitro *it is possible to produce infectious retroviral particles carrying a high-level expression cassette that completely prohibits production of infectious retroviral particles by conventional methods.

We produced an expression cassette comprising a strong enhancer/promoter, an optimised intron, the GFP open reading frame and a strong polyadenylation signal. This cassette was cloned into both a conventional MMLV retroviral vector and a vector designed to allow *in vitro *transcription of the retroviral genome by T7 RNA polymerase.

When the conventional retroviral vector was transfected into packaging cells, the expression cassette drove strong GFP expression, but no infectious retrovirus was produced. Introduction of the *in vitro *produced uncapped retroviral genomic transcript into the packaging cells did not lead to any detectable GFP expression. However, infectious retrovirus was easily recovered, and when used to infect target primary human cells led to very high GFP expression – up to 3.5 times greater than conventional retroviral LTR-driven expression.

**Conclusion:**

Retroviral vectors carrying an optimized high-level expression cassette do not produce infectious virions when introduced into packaging cells by transfection of DNA. Infectious retrovirus carrying the same cassette is readily produced when packaging cells are transfected with *in vitro *transcribed retroviral genomic RNA. The applications of this technique are not limited to producing the higher levels of transgene expression demonstrated here. For example, novel reporters with alternatively spliced exon-intron configurations could readily be transduced into virtually any cell. Furthermore, because the *in vitro *transcripts are not translated within the packaging cells, retroviruses carrying genes lethal to the packaging cells can also be produced.

## Background

**Figure 1:**
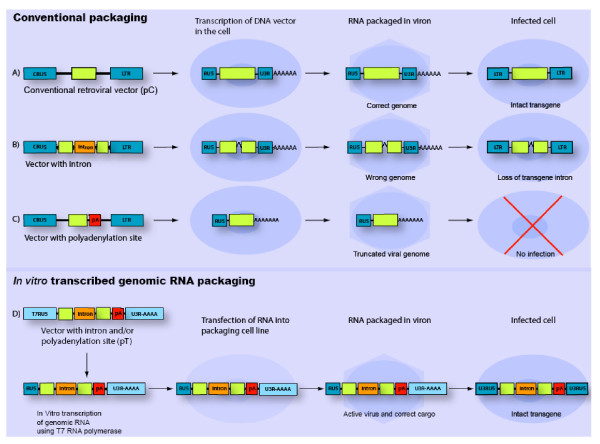
**Production of retroviruses by conventional packaging cell transcription or *in vitro *transcribed genomes**. **A) **Retroviruses are produced by transfecting vector plasmids into a packaging cell line (e.g. 293T-based Phoenix cells) that expresses requisite viral structural proteins. *Cis*-sequences in the LTR promote transcription and editing of the retroviral RNA genome which is then exported to the cytoplasm for assembly into a viral particle. Mature virions bud from the surface and can infect target cells. **B) **If an intron is embedded in the retroviral sequence it is removed by the nuclear splicing prior to packaging, such that the packaged retroviral genome lacks the intron. **C) **A retroviral vector incorporating an additional polyadenylation signal (pA) will yield an unproductive truncated viral genome. **D) **A new approach is to synthesize the retroviral genomic transcript *in vitro *and transfect it into the packaging cell cytoplasm where it is directly packaged into a virus particle. In the pT system vector, the promoter/enhancer (U3) sequence in the 5'LTR is replaced with the promoter sequence for bacteriophage T7 RNA polymerase, enabling *in vitro *transcription of the retroviral genomic RNA. Avoidance of nuclear functions allows packaging of retroviral RNA incorporating various editing signals (introns and polyadenylation signals) and subsequent transfer into target cells.

Retroviral vector systems are routinely used as delivery vehicles for efficient and stable gene transfer into mammalian cells [1]. Since their inception more than 20 years ago, retrovirus vectors have been developed to transfer various genetic elements for varied purposes: Stable expression of cDNA for gene studies and therapy; efficient expression of short hairpin RNA (shRNA) to trigger RNA interference; integration of splice donor sequences for gene trapping; generation of gene reporter cell lines [2,3]. In spite of the accommodating nature of the retroviral genome, there are inherent limitations in the retroviral life cycle that constrain vector design and impede full utilization of the system [4]. The retroviral genome must be produced by RNA polymerase II in the nucleus of the packaging cell, edited (capped and polyadenylated) and transported to the cytoplasm for assimilation into a mature virion at the plasma membrane. Hence sequences that interfere with nuclear export or express products detrimental to packaging cell function or survival will not yield virus. Extraneous post-transcriptional processing signals present in the vector sequence will produce incomplete genomic transcripts (e.g. polyadenylation signal) or be removed prior to packaging and not transferred (e.g. introns) [5,6] (Figure 1A–C). Frequently, vectors are designed with an internal promoter to drive high transgene expression which leads to "promoter interference" with the retroviral long-terminal repeat (LTR) and severely compromises viral titer [7,8].

## Results and Discussion

In spite of the popularity of retroviral vectors, little has been done to address these limitations. We recently developed a simple approach that dramatically expands the range of genetic elements transferable by retroviral vectors [9]. The system employs *in vitro *generated RNA to nucleate retroviral virions, completely circumventing packaging cell genomic transcription and translation (Figure 1D).

To demonstrate the utility of this innovation, we endeavoured to create a high expression retroviral vector using a known optimal constellation of genetic elements predicted to be incompatible with current systems. Indeed we show that a retroviral vector with substantially higher transgene expression in primary human cells can be constructed using the *in vitro *transcribed retroviral genomic RNA approach, while the same gene cassette prohibited the production of virus by the conventional system.

**Figure 2:**
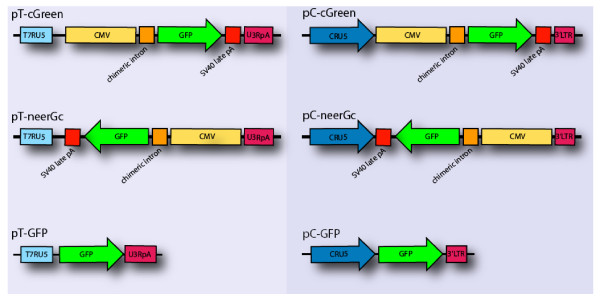
**Schematics of the retroviral vectors**. Standard retroviral vectors were based on the pC-GFP vector [12]. pC-cGreen and pC-neerGc, carry an additional optimized GFP expression cassette comprising the CMV immediate early promoter, a chimeric intron and SV40 late pA, in opposite orientations. The pT-GFP vector has a T7RU5 composite 5'-LTR and modified 3'LTR where the U5 region has been replaced by a poly-A tract for *in vitro *transcription of vector genomic RNA [9]. pT-cGreen and pT-neerGc each contain a CMV expression cassette as above.

A series of retroviral vectors were constructed to carry a standard strong GFP expression cassette comprising EGFP cDNA flanked by an optimized intron, and a potent late SV40 polyadenylation signal under the control of the cytomegalovirus immediate early (CMV IE) promoter [10]. This expression cassette was cloned in both orientations into a standard retroviral vector (pC) or into a retroviral vector designed to allow *in vitro *transcription of the retroviral genomic RNA (pT) (Figure 2). pT is similar to conventional retroviral vectors except that the U3 region of the 5' LTR has been replaced by the binding site for bacteriophage T7 RNA polymerase, positioned so that transcription initiates at the correct nucleotide. The U5 region of the 3'LTR, which contains the natural polyadenylation signal, is replaced by 40 A's to mimic a polyA tail followed by a site for the restriction enzyme MluI to allow linearisation of the vector. As controls, we used the GFP open reading frame alone (without the additional promoter, intron or polyadenylation site) cloned into both conventional (pC-GFP) and T7-transcribed (pT-GFP) retroviral vectors. On integration into the target cell genome, these vectors are expected to produce essentially identical sequences.

**Figure 3:**
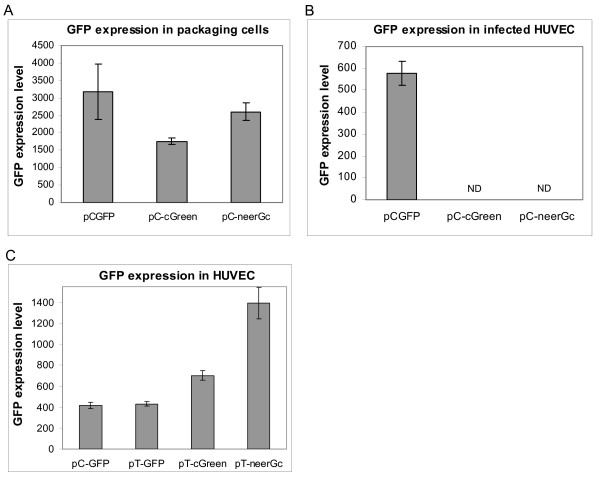
**A) Packaging cell expression**. Packaging cells were transfected with equal amounts of pC-GFP, pC-cGreen and pC-neerGc retroviral vector plasmids. GFP expression was quantified in single cells at 24 hours by flow cytometry. **B) Expression levels in infected cells**. At 18 hours post-transfection, conditioned medium was harvested from the packaging cells and used to infect human umbilical vein cells (HUVEC). GFP expression was quantified in single cells at 48 hours post-infection by flow cytometry. pC-GFP infected cells expresses strong GFP levels, while neither the pC-cGreen nor the pC-neerGc retroviral vector produced detectable viral infection. ND: none detected. **C) A high expression retroviral vector using *in vitro *transcribed genomes**. Early passage primary endothelial cells (HUVEC) were infected for 24 hours using serial dilutions of virus generated by the pC-GFP, pT-GFP, pT-cGreen and pT-neerGc vectors. The GFP expression level of individual cells harboring single viral integrations was measured by flow cytometry at 48 hours post-infection. GFP expression in the proviruses driven by the optimized CMV promoter/intron/pA cassette (pT-cGreen, pT-neerGc) is substantially higher than that obtained by LTR-promoter dependent expression (pC-GFP, pT-GFP).

When transfected into packaging cells, the standard retroviral vectors (pC) produced high levels of GFP expression as measured by flow cytometry (Figure 3A). However, the presence of the internal CMV-expression cassette completely abrogated production of infectious virus (Figure 3B). The complete loss of infectious virus in both pC-cGreen and pC-neerGc is primarily due to the presence of the strong, bidirectional SV40 polyadenylation site [11]. However, competition between the internal CMV promoter and the 5' LTR CMV promoter is also likely to contribute to the loss of titer [7].

In contrast, *in vitro *transcription system based vectors (pT) carrying the same CMV expression cassette readily generated infectious virus following transfection of *in vitro *transcribed RNA genomes into packaging cells. The pT vectors produced no detectable GFP expression in the packaging cells, due to the lack of requisite translation signals (e.g. 5' cap) on the RNA transcripts, a notable advantage of the system (not shown;[1]). Primary human umbilical cord endothelial cells (HUVEC) were readily infected with viral supernatants generated by the pT-vectors carrying the strong GFP expression cassette (pT-cGreen and pT-neerGc) and expressed markedly higher (up to 3.5-fold) levels than obtained with the conventional LTR-driven (pC-GFP, pT-GFP) vectors (Figure 3C). The reverse orientation cassette (pC-neerGc, pT-neerGc) consistently gave higher expression than the forward orientation cassette (pC-cGreen, pT-cGreen) (Figure 3A, C). The reason for this is unknown but presumably reflects a more favorable context for CMV-driven expression proximal the 3' LTR compared to near the packaging sequence (Ψ).

## Conclusion

In this report we demonstrate how *in vitro*-transcribed retroviral RNA genomes can be utilized to transfer a high-level expression cassette which includes genetic elements not compatible with commonly used retroviral packaging systems. Using this cassette we observe an increase in expression level of up to 3.5 times in primary human cells, reflecting the provision of an intron and the use of strong enhancer/promoter and polyadenylation signals. The ability to circumvent packaging cell transcription, post-transcriptional editing and translation of the vector genome, while maintaining assembly into virions, effectively addresses the shortcomings of current retroviral systems. Thus, this approach is based on standard molecular biology techniques, yet allows virtually any genetic sequence constellation to be manifested by the integrated retroviral provirus: Strong enhancers/promoters, introns and polyadenylation signals for boosting gene expression; gene reporters that encompass multiple exon-introns; post-transcriptional signal sequences to improve gene trapping; cytotoxic gene expression or shRNA expression cassettes that target essential genes.

## Methods

### Vectors

Vectors pT-cGreen/neerGc (L311/L312) were based on vector L139 pT7RU5mcsSIN, a derivative of LB-G [9] in which the GFP has been removed and the 3'LTR replaced with a self-inactivating 3'LTR (SIN) from the vector pTra [2]. L139 thus carries in the following order: A T7 site fused to the transcriptional start site of the MMLV RU5 region of the LTR, an extended packaging sequence (Ψ), a multiple cloning site and a self-inactivating 3'LTR followed by forty A residues. Vectors pC-cGreen/neerGc (L309/L310) were based on vector L060 pCRU5 Puro2Amcs, a derivative of p96.7 [12]. This vector carries a composite CMV promoter fused to the transcriptional start site of the MMLV RU5 region of the LTR, an extended packaging sequence (Ψ), multiple cloning site and 3'LTR. Vector pCI (Promega) contains a CMV promoter, an optimized chimeric intron and an SV40 polyadenylation site selected to drive strong constitutive expression [10]. A T7 polymerase initiation site within the expression cassette of pCI was removed by deleting the sequence between the unique NheI site (filled) and the ScaI site 22 bp upstream (pCIdeltaT7). An EcoRI/NotI fragment from pCGFP [13] encompassing the GFP coding sequence was then cloned into the corresponding sites of pCIdeltaT7. The complete CMV-intron-GFP-SV40pA cassette was excised with BglII and ClaI, blunt ended and cloned into L060 (XhoI, EcoRI, blunted) and L139 (PmeI). Both orientations of insert were obtained, producing pT-cGreen/neerGc and pC-cGreen/neerGc.

### Genomic RNA production

To generate the uncapped retroviral genomic transcripts, MluI-linearized pT-vector DNA was transcribed with T7 polymerase (MEGAscript, Ambion, Austin, TX). RNA was quantified (ND-1000 spectrophotometer, NanoDrop Technologies, Wilmington, DE) and the quality checked by denaturing agarose gel electrophoresis [14] (data not shown).

### Transfection and retroviral transduction

Standard retroviral vectors (pC) were transfected into 293-based Phoenix A packaging cells by the standard protocol [12,15] which for pC-GFP typically produces titers of around 1 × 10^7 ^gfu/ml for infection of primary human endothelial cells.

The *in vitro *transcribed genomic RNA was introduced into the packaging cells using the cationic lipid DMRIE-C (Invitrogen, San Diego, CA). The RNA was mixed with lipid at a ratio of 1 μg RNA to 3.5 μl DMRIE-C in a final volume of 250 μl of serum-free OptiMEM (Invitrogen, San Diego, CA) and incubated at room temperature for 20 minutes. Subconfluent (80%) Phoenix-A packaging cells in poly-lysine coated 12-well plates were prepared by rinsing with serum-free medium and 360 μl OptiMEM was added per well. The RNA-lipid mixture was added dropwise to the cells and incubated for 2 hours before the cells were washed and incubated in complete medium. pT virus-containing medium (1 ml) was harvested 18 hours later. Titers were typically approximately 1 × 10^5 ^gfu/ml for infection of primary human endothelial cells [9].

Primary human umbilical vein endothelial cells (HUVEC, passage 1–3; Cambrex, Rockland, ME) were cultured according to the supplier's recommendations. Cells were infected as described (e.g. plated at 40,000/12-well; 5 μg/ml protamine sulfate) [15]. Viral supernatants were diluted in medium to obtain a multiplicity-of-infection yielding 10–20% GFP-positive cells corresponding to single viral infections. Typical dilutions used were 1/100 (pC-GFP) or 1/3–1/9 (pT vectors). GFP expression measurements were performed on a FACSCalibur cytometer (Becton Dickinson, San Jose, CA) and analysis was carried out using Flowjo [16].

## Authors' contributions

All authors participated in the overall study design and writing of the manuscript. MB/DM conducted the experiments in the laboratory of JBL. All authors have read and approved the final manuscript.
